# The first national pediatric immune thrombocytopenia registry in Iran: research protocol and preliminary study results

**DOI:** 10.1186/s12913-024-11102-z

**Published:** 2024-05-28

**Authors:** Sharareh Kamfar, Sabahat Haghi, Vahide Zeinali, Parastoo Molaei Tavana, Reza Arjmand, Fatemeh Malek

**Affiliations:** 1 Pediatric Congenital Hematologic Disorders Research Center, Research Institute for Children’s Health, Shahid Beheshti University of Medical Sciences, Tehran, Iran; 2 Department of Hematology and Oncology, School of Medicine, Alborz University of Medical Sciences, Karaj, Iran; 3 Research Institute for Children’s Health, Shahid Beheshti University of Medical Sciences, Tehran, Iran; 4 Department of pediatrics, Loghman Hakim Hospital, Shahid Beheshti University of Medical Sciences, Tehran, Iran; 5 Department of Pediatrics, School of Medicine, Alborz University of Medical Sciences, Karaj, Iran

**Keywords:** Immune thrombocytopenia, Registries, Minimum data set, Iran

## Abstract

**Background:**

Disease registries are comprehensive databases that record detailed information on patients diagnosed with specific conditions, providing valuable insights into their diagnosis, treatment, and outcomes. This study aims to describe the pilot phase of the national pediatric Immune Thrombocytopenia(ITP) registry (NPITP) in Iran, serving as the inaugural interpretive report.

**Methods:**

This patient-centered software system was implemented as a national program across multiple pediatric centers in Iran. Several focus groups were conducted to establish a minimum data set (MDS) comprising six main classes, 14 sub-classes, and 187 data elements. Following expert consensus on the final data set, a web-based software tool was developed by the dedicated IT team, accessible online and offline via https://disreg.sbmu.ac.ir/q/ITP.html. The registry included children aged between two months and 18 years with a platelet count below 100 × 10^9^/L, based on predefined inclusion criteria.

**Results:**

Within a four-month period, a total of 60 ITP patients were registered, including 41 (68.3%) newly diagnosed cases, 68 (13.6%) persistent cases, and 14 (23.3%) with chronic ITP. The mean age of the registered patients was 55.93 ± 9.72 months. The most frequently observed bleeding symptoms were petechiae (68.3%), purpura (51.6%), and ecchymosis (13.3%). Among the newly diagnosed patients, 20 (33.3%) received intravenous immunoglobulin (IVIG), 17 (28.3%) were treated with prednisolone, and 17 (28.3%) received combined IVIG and steroid therapy. Of all patients, 40 (66.7%) demonstrated a complete response to treatment, while 16 (26.7%) exhibited a partial response. Four patients (6.7%) remained unresponsive to therapy. Treatment-related complications, such as Cushing’s syndrome, edema, weight gain, hirsutism, and mood disorders, were reported in 10 patients (16.6%). However, the majority of patients (81.7%) did not experience therapy-related complications.

**Conclusion:**

The pilot phase of the NPITP registry successfully implemented a web-based software tool for data collection, aiming to enhance the quality of care, facilitate clinical research, and support health service planning in the future.

## Introduction

Immune thrombocytopenia(ITP) is a commonly acquired bleeding disorder in children, with an annual incidence rate of 5 to 10 per 100,000 children [1]. Typically, ITP is a self-limiting disease that resolves within six months, and children generally experience mild bleeding symptoms such as bruising, petechiae, and purpura [2]. The exact mechanisms responsible for platelet destruction and reduced platelet production in ITP are still not fully understood [3]. ITP patients are classified into three types based on the duration of the disease: newly diagnosed (< 3 months), persistent (3–12 months), and chronic (> 12 months) [4, 5]. Diagnostic evaluation and treatment approaches differ between pediatric and adult ITP, with reports indicating that adult ITP is often chronic and more severe than pediatric cases [6]. Platelet reduction can predispose patients to various bleeding events, ranging from mild skin bruises to life-threatening intracranial hemorrhage [7, 8]. The 2019 guidelines from the American Society of Hematology recommend a “Wait and watch” approach for newly diagnosed ITP in children without symptoms or with only cutaneous bleeding manifestations. Non-life-threatening bleeding events can be managed with a short course of corticosteroids, intravenous immunoglobulin (IVIG), or anti-D immunoglobulin therapy [7].

It is important to note that there is currently no consensus guideline in our country, making it essential to provide data on the relative safety and effectiveness of different therapeutic approaches. This will contribute to improving the quality of our healthcare delivery system and promoting resource-saving. IVIG is one of the most commonly used therapeutic options for childhood ITP, with an estimated consumption rate of approximately 1500 kg in the Islamic Republic of Iran in the previous year, which has been steadily increasing over the past decade [8].

Due to insufficient coding and organized data collection for rare diseases, patient information is rarely identifiable in hospital information systems. However, significant progress has been made in understanding ITP over the past decade through data collection from large cohorts of patients. This includes the clinical course of the disease, disease prevalence and incidence, risk factors for bleeding, treatment strategies, and adverse outcomes. Despite these advancements, many questions about ITP remain unanswered. In 1996, the American Society of Hematology released the first guidelines for diagnosing and managing patients with ITP, which have become the standard references for clinicians managing the disease [9, 10]. Furthermore, it is crucial to emphasize shared decision-making in addressing treatment-related issues. Patient registries are valuable sources of data in various diseases, including pediatric immune thrombocytopenia (ITP), and can provide comprehensive knowledge for the assessment and management of critical disease features [11]. They also facilitate effective communication and data sharing to enhance the disease management process. In 2020, against the backdrop of debates surrounding ITP treatment in Iran and the absence of a practice guideline, the national pediatric ITP registry (NPITP) was established for the first time in the country. The primary objective of the NPITP was to develop a patient-centered national guideline based on the best available evidence, focusing on the diagnosis and management of the disease. The registry aims to achieve the following objectives:

- Determine the primary medical conditions, age at disease diagnosis, and family medical history.
- Identify clinical, para-clinical, and laboratory findings.
- Assess disease complications and bleeding manifestations.
- Classify disease status (newly diagnosed, resistant, chronic).
- Evaluate the clinical course and disease management.
- Conduct bone marrow evaluations for ITP diagnosis.
- Identify treatment options used for ITP patients and their outcomes.
- Determine patients’ response to treatment.
- Assess treatment-related complications.
- Investigate the relationship between platelet count and bleeding severity in this disease.

## Design and methods

### Registry design

Mofid Children’s Hospital has taken a pioneering role in the design of a national pediatric ITP registry in Iran. The establishment of this national, multi-centric registry in September 2020 aims to collect essential information on the disease, including laboratory and clinical data, from children aged between two months and 18 years with a diagnosis of acute, persistent, and chronic ITP and a platelet count of less than 100 × 10^9^/L [12]. The registry’s primary objective is to provide continuous follow-up for these children, as necessary.

The NPITP registry initially began its operations at Mofid Children’s Hospital and two other participating centers, namely Baqiyatallah Hospital in Tehran and Imam Ali Hospital in Karaj City. The project intends to collaborate with additional centers to establish a national guideline. During the pilot phase of the NPITP registry, which was conducted to identify any issues or shortcomings, particular emphasis was placed on ensuring the accuracy of data entry. Furthermore, the pilot phase validated the data collection system, enhanced the tools for registry-specific data collection, and assessed the accuracy and completeness of the registry.

### Ethical considerations

Given the registry nature of our study, involving solely data registration without participant interaction, consent was considered unnecessary. All the procedures performed in the study were approved by Ethics Committee of Shahid Beheshti University of Medical Sciences (IR.SBMU.RICH.REC.1399.021) and the 1964 Helsinki declaration and its later amendments.

### Registry process

**Fig. 1:**
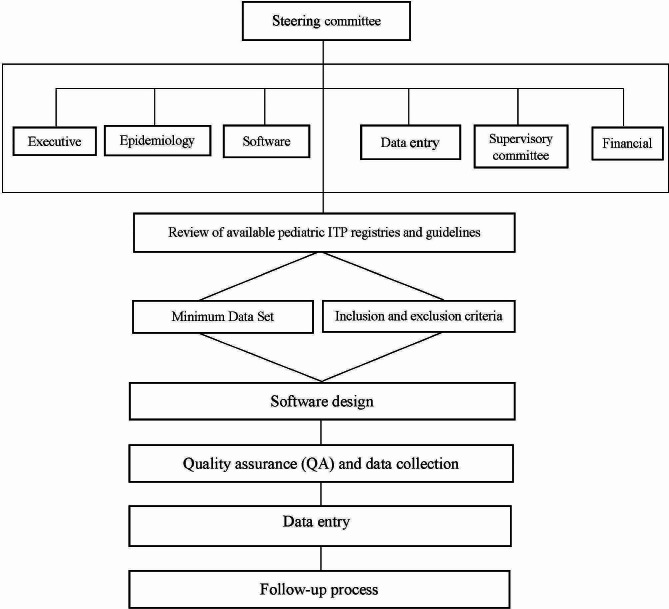
The key steps in planning NPITP registry

The NPITP registry utilized a systematic approach consisting of nine steps (Fig. 1) as follows:

- Formation of Expert Groups: Expert groups were established to effectively address the objectives of the registry.
- Review of Existing Registries and Guidelines: A comprehensive assessment of available pediatric ITP registries and guidelines was conducted.
- Development of Data Elements and Minimum Data Set (MDS): The registry team worked on defining the necessary data elements and establishing a minimum data set.
- Identification of Eligible Patients: Criteria were established to identify patients who met the inclusion criteria for the registry.
- Software Design: A dedicated team of experts designed the software to meet the specific requirements of the registry.
- Quality Assurance and Data Collection Assessment: An evaluation of quality assurance measures and data collection procedures was conducted to ensure reliable and accurate data.
- Data Entry: Trained data entry professionals were responsible for entering patient records into the software.
- Follow-up Process: A systematic process was implemented to ensure the ongoing follow-up of patients within the registry.
- Data Analysis: The collected data was analyzed using statistical techniques and procedures, carried out by a team of epidemiologists.

### Expert groups formation

To effectively manage the registry, seven groups were established with specific responsibilities:

- Steering Committee: Comprising pediatric Hematologists-oncologists, this committee played a crucial role in discussing the registry’s objectives, validating data elements, and approving the minimum data set. Additionally, the steering committee oversaw the functioning of sub-committees.
- Executive Committee: Responsible for managing the registry team members, this committee proposed changes and implemented policies and procedures within the registry.
- Epidemiologists Team: This team consisted of experts who determined the statistical techniques and procedures required for evaluating and analyzing the registry-related data.
- Software Designer Team: Comprising a board of experts, this team designed the software to cater to the specific needs and purposes of the registry.
- Supervisory Committee: Supervisors within this committee monitored and coordinated the activities of the registry, including staff and experts.
- Data Entry Professionals: These trained experts were entrusted with the responsibility of accurately entering patient data into the software.
- Financial Sponsor Group: This group assumed the financial responsibility for the clinical investigation. The pediatric ITP registry received funding from the Disease Registry Center of Shahid Beheshti University of Medical Sciences.

### Review of available pediatric ITP registries and guidelines

The establishment of the American Society of Hematology guidelines on immune thrombocytopenia (ITP) aims to provide valuable support for decision-making in the management of patients with ITP [7]. Additionally, numerous pediatric ITP registries offer national, international, or global data on the prevalence, diagnosis, and management of this disease (Table 1). One prominent organization contributing to these efforts is the Intercontinental Cooperative ITP Study Group (ICIS), which was founded in 1997. ICIS regularly organizes meetings and has established various registries, including the Pediatric and Adult Registry on Chronic ITP (PARC-ITP). Since 2004, PARC-ITP has served as a comprehensive, worldwide multi-center registry dedicated to prospectively collecting data on individuals below 16 years of age and adults with newly diagnosed ITP [13, 14]. In France, the OBS’CEREVANCE registry is a prospective national registry that focuses on registering data from children below 18 years of age with chronic ITP [14, 15]. The UK Pediatric ITP registry was established to gather information on newly diagnosed or chronic ITP in children below 18 years of age in the UK, while also monitoring those with chronic ITP [14, 16]. The Norwegian ITP registry, established in 2017, serves as both a retrospective and prospective clinical registry, with a specific emphasis on collecting data from children and adults with ITP [14]. These registries collect crucial information on bleeding severity, platelet counts, complications associated with ITP treatments, and adverse events during follow-up. Overall, these pediatric ITP registries and guidelines play a pivotal role in advancing our understanding of ITP, facilitating informed decision-making, and improving patient management.

**Table 1:** clinical registries and guideline used for creating minimum data set

Registry	Patients
The American Society of Hematology guidelines	Children and adults
Pediatric and Adult Registry on Chronic ITP (PARC-ITP)	Children (< 16 years) and adults with newly diagnosed ITP
UK-children ITP registry	Children (< 18 years) with newly diagnosed or chronic ITP
OBS’CEREVANCE	Children (< 18 years) with chronic ITP
Norwegian ITP registry	Children and adults with ITP

### Identification of eligible patients

In order to identify patients meeting the established inclusion criteria, a careful selection process was implemented. The primary diagnosis of immune thrombocytopenia (ITP) served as the basis for inclusion. Specifically, children between the ages of two months and 18 years, with a platelet count lower than 100 × 10^9^/L, were considered eligible. It is important to note that ITP was designated as an exclusionary diagnosis, as it was crucial to evaluate treatment response and observe platelet count trends independent of other underlying conditions. Consequently, patients with thrombocytopenia related to other diseases (such as HIV infection or hepatitis C infection) were excluded from participation.

### Development of data elements and Minimum Data Set (MDS)

To ensure effective reporting and meet the information requirements of the NPITP registry, a minimum data set (MDS) comprising various data elements was meticulously constructed. Each data element was selected in a stepwise manner to align with the registry’s objectives. A comprehensive evaluation was conducted to determine the essential and desirable elements for inclusion in the primary data set. Initially, clinical data from patients’ medical records at Mofid Children’s Hospital were utilized to create the primary data set. Subsequently, the data set was further refined and expanded in accordance with the guidelines established by the American Society of Hematology (ASH) and other ITP-related registries worldwide. The inclusion of practical and usable data collection tools required unanimous agreement among all members of the registry team. Therefore, regular group meetings were held to facilitate discussions and ensure the selection of accurate data elements.

#### Software design

To ensure complete compatibility with registry purposes, the initial requirement was to establish a consensus on the set of data elements. Subsequently, a web-based software solution with specific features was meticulously designed to guarantee accurate data entry within the NPITP registry. Given the significant problem of duplicate patient records in medical systems, the ITP software was developed to prevent such occurrences effectively.

Compliance with privacy regulations governing the NPITP registry dictated that only authorized individuals could access a patient’s medical history. Consequently, we categorized data accessibility into three distinct groups: project managers, executive managers, and registry experts. It is important to highlight that the access privileges of registry experts were complemented by comprehensive training on data quality issues and patient data entry records within the registry software.

### Assessment of quality assurance and data collection

Prior to data collection, comprehensive quality assurance and data quality assessment procedures were implemented to enhance data integrity. This involved the improvement of data quality, the establishment of standardized protocols, the implementation of robust data management systems, and staff training [17, 18]. Additionally, an evaluation was conducted to assess the quality of data collection methods and the efficacy of data storage techniques in the database. Specific measurements, such as validity, reliability, accuracy, completeness, comparability, relevance, timeliness, effectiveness, and consistency, were employed to scrutinize the collected data. The outcomes of these evaluations facilitated data analysis, knowledge development, and continuous monitoring of the registry protocol, thereby contributing to the systematic organization of protocols within the ITP registry.

Scientific studies have consistently demonstrated that enhanced standardization of registry protocols and checklists significantly reduce patient harm and ensure consistent and accurate treatment decisions [19]. In response to the need for comprehensive patient information obtained from medical records, a tailored data management system was designed specifically for the purposes of the ITP registry. This system enables more precise diagnoses, prognoses, and facilitates the formulation of effective health policies based on medical registry data.

### Data entry

Following the design of the software, a pilot phase was initiated to evaluate the accuracy of the registry system. In this phase, the medical history of 60 patients was recorded, with only one hematologist responsible for completing the forms. Subsequently, data entry specialists were tasked with inputting the collected data into the software. This process allowed for a meticulous assessment of the software’s accuracy and functionality.

### Follow-up process

Consistent follow-up plays a pivotal role in patient registries, making periodic patient follow-up an essential requirement [20]. The patients’ follow-up plan is determined by the clinician’s decision, and during these visits, both laboratory and clinical data are collected.

### Data analysis

To effectively report information in the NPITP registry, various features were employed in a specially designed software system. For statistical analyses, IBM SPSS Statistics ver.23 (IBM, Armonk, NY, USA) was utilized in this study. Quantitative variables were presented as mean ± standard deviation, while qualitative variables were expressed as numbers (percentages). For comparing patients in the Acute/persistent ITP and Chronic ITP groups, the Mann-Whitney U test and Fisher’s exact test were applied. Moreover, Spearman’s correlation rank coefficient was used to assess correlations between quantitative variables. Ultimately, the findings of the NPITP registry will be documented in written form.

## Results

**Fig. 2:**
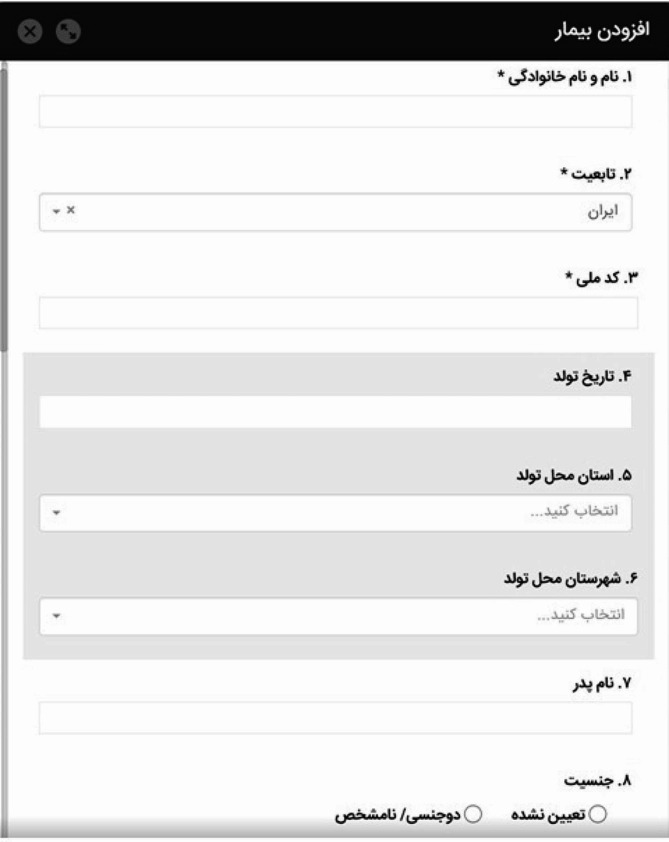
A screenshot of demographic data in the NPITP registry Software

Based on the experts’ consensus on the final data set, an engineering team developed web-based software, accessible both online and offline, at https://disreg.sbmu.ac.ir/q/ITP.html. The majority of data items in the ITP data set could be selected from a drop-down list. Notably, the registration process required mandatory information such as the individual’s name, family name, ID code, and ethnicity to prevent duplicate records. Figure 2 depicts a screenshot of the NPITP software. However, this study presents the initial version of the pediatric ITP dataset, encompassing six main classes, 14 sub-classes, and 187 data elements, as outlined in Table 2.

**Table 2:** The main classes and subclasses of MDS in NPITP registry system

Main class	Sub-class	Data element
Administrative data	Demographic data	Name*, Family name*, ID Code*, Date of birth, Age, Gender, Father’s name, Ethnic, Place of birth, Phone, Center of history taking
	Patient information at the time of admission	First date of admission, date of diagnosis, age at diagnosis
Past medical history	Patient’s medical history	History of infection (viral and/or H. pylori), collagen vascular disease, immune disorder, Evans syndrome, malignancies, surgery, blood product injection
	Family medical history	History of collagen vascular disease, hereditary bleeding disorders, and thrombocytopenia
	Medication history	Drug-uptake history (warfarin, aspirin, direct oral anticoagulants (DOACs), low molecular weight heparins (LMWH), non-steroidal anti-inflammatory drugs (NSAIDs))
Clinical diagnostic tests	Laboratory test	CBC with Differential, immunoglobulins blood test, complement blood test (C3, C4, CH50), immunological tests (HBs Ab, HBs Ag, HIV Ab, direct antiglobulin test, lupus anticoagulant, anti-nuclear Ab, anti-Cardiolipin Ab, anti-dsDNA
	Medical imaging	CT scan findings, MRIs, ultrasound scan
	Bone marrow evaluation	Bone marrow aspiration and bone marrow biopsy
Disease status	Disease classification	Acute, persistent, and chronic ITP
	Signs and symptoms	Petechiae, purpura, hematuria, hematochezia, ecchymosis, epistaxis, Hematemesis, melena, menorrhagia, and intracerebral hemorrhage
Treatment	Medications and treatment methods	IVIg, prednisolone, anti D Immunoglobulin, methylprednisolone, dexamethasone, dapsone, azathioprine, rituximab, cyclosporine, mycophenolate, cyclophosphamide, vinca alkaloids, thrombopoietin agonist, platelet transfusion, plasmapheresis, and other (s)
	Response to treatment	Partial response, complete response, unresponsive
	Treatment-related complications	weight gain, osteoporosis, Cushing’s syndrome, diabetes, blood clots, hirsutism, thrombosis, mood disorders, pseudotumor cerebri, edema, transaminitis, cataracts, myelofibrosis, hypertension, no complications, death
Follow-up	Patient status at least until 2 weeks after discharge	Drug-uptake history, laboratory test, BMB evaluation, disease status, symptoms, types of treatment, treatment response status, and treatment complication.

During the pilot phase of this registry, collaborative centers registered a total of 60 patients diagnosed with ITP. The number of enrolled patients varied across centers, with 40 patients registered at Mofid Children’s Hospital, 11 at Baqiyatallah Hospital, and 9 at Imam Ali Hospital. Among the registered patients, 39 were male and 21 were female. The average age at the time of diagnosis was 55.93 ± 9.72 months. Table 3 provides an overview of the demographic and clinical characteristics of the 60 ITP patients, including 45 cases of acute/persistent ITP and 14 cases of chronic ITP.

**Table 3:** Demographic and clinical characteristics of the registered patients in the Iranian NPITP registry

Parameters	Acute/persistent ITP (*n* = 45)	Chronic ITP (*n* = 15)	*P*-value
**Sex (male)**	32 (71.11%)	7 (46.67%)	0.12
**Age of onset (years)**	53.40 ± 47.73	63.53 ± 56.36	0.71
**History of infection**	7 (15.56%)	1 (6.66%)	0.09
**Platelet count at diagnosis ×10** ^**3**^ **/µL**	39391.37 ± 89147.35	20423.40 ± 25672.34	0.41
**Clinical symptoms**			
Petechiae	32 (71.11%)	9 (60%)	0.52
Purpura	20 (44.44%)	11 (73.33%)	0.07
Epistaxis	3 (6.66%)	2 (13.33%)	0.59
Ecchymosis	5 (11.11%)	3 (20%)	0.40
Gum bleeding	3 (6.66%)	2 (13.33%)	0.36
Menorrhagia	0	1 (6.66%)	0.25
**Treatments and medications**			
IVIg	18 (40%)	2 (13.33%)	0.001
Prednisolone	16 (35.56%)	1 (6.66%)	
Methylprednisolone	1 (2.22%)	0	
Dexamethasone	1 (2.22%)	0	
Azathioprine	0	1 (6.66%)	
**Response to treatment**			
Partial response	14 (31.11%)	2 (13.33%)	0.24
Complete response	29 (64.44%)	11 (73.33%)	
Unresponsive	2 (4.44%)	2 (13.33%)	
**Complications and outcomes**			
Weight gain	0	2 (13.33%)	0.05
Cushing’s syndrome	1 (2.22%)	5 (33.33%)	0.003
Hirsutism	0	2 (13.33%)	0.59
Mood disorders	1 (2.22%)	0	1.00
Pseudotumor Cerebri	0	0	-
Edema	0	1 (6.66%)	0.25
Death	0	0	-
No complications	43 (95.56%)	7 (46.66%)	< 0.001

The results revealed no correlation between age at diagnosis and the type of ITP (*P* = 0.26). Furthermore, no registered patients had a family history of thrombocytopenia. Of the cases, eight (13.3%) had a history of previous infection, comprising seven newly diagnosed ITP cases and one chronic case. The mean platelet count at diagnosis was 34649.38 ± 78433.1, and the mean hemoglobin level was 11.55 ± 2.28 g/dl. Among the registered patients, 41 (68.3%) were newly diagnosed with ITP, 68 (13.6%) had persistent ITP, and 14 (23.3%) had chronic ITP. Bone marrow aspiration (BMA) was performed on 38 ITP patients (63.3%), including 25 newly diagnosed cases, 11 chronic cases, and two persistent cases. The BMA results indicated normal cellularity without significant findings. Megakaryocyte counts were of particular interest; however, they were not consistently recorded in most cases. Only four out of the 15 patients with chronic ITP underwent bone marrow biopsy (BMB), among whom three showed no increase in the number of megakaryocytes.

The results demonstrated no statistically significant gender differences across all categories of immune thrombocytopenia (ITP) (*P* = 0.10). Moreover, a statistically significant correlation was observed between the type of ITP and bleeding manifestations (*P* = 0.002). The most commonly reported bleeding symptoms among patients were petechiae, purpura, and ecchymosis. Specifically, clinical presentations included petechiae in 42 patients (70%), purpura in 33 patients (55%), ecchymosis in 8 patients (13.3%), epistaxis in 5 patients (8.3%), gingival bleeding in 5 patients (8.3%), and menorrhagia in 1 patient (1.7%). However, 8 out of the total 60 patients (13.3%) exhibited no significant symptoms. Notably, the results indicated no significant correlation between platelet count and bleeding manifestations (*r*= -0.11; *P* = 0.40).

Among the cohort, 20 newly diagnosed ITP patients (33.3%) received IVIG, while 17 patients (28.3%) were treated with prednisolone alone, and 17 patients (28.3%) underwent combined IVIG and steroid therapy. The remaining patients received methylprednisolone, dexamethasone, or azathioprine as part of their treatment regimen. Of the total, 40 patients (66.7%) achieved a complete response to therapy, while 16 patients (26.7%) exhibited a partial response. Four patients (6.7%) did not respond to the treatment. Adverse events associated with treatment, such as Cushing’s syndrome, edema, weight gain, hirsutism, and mood disorders, were reported in 10 patients (16.6%). However, the majority of patients (81.7%) did not experience any complications due to therapy.

## Discussion

Given the low reported incidence rate of ITP and its variable methodology, it becomes imperative to accurately estimate its incidence in order to determine its impact on public health [8]. Patient registries play a crucial role in understanding the epidemiology and management of rare diseases. By providing systematic data on disease-related events, these registries bridge information gaps in healthcare systems. Additionally, the availability of electronic health records enables the evaluation of other data resources for diseases like ITP.

Data sharing is an integral aspect of research that enhances the scientific efficiency of collected data while preventing duplicated research [21]. However, it also raises ethical and legal concerns. To address these concerns, privacy rules have been established, providing pathways for registries to use patient information responsibly. In line with this, we have successfully established the NPITP registry in Iran, marking the first of its kind.

The implementation of the NPITP registry involved the formation of a group of experts who developed the registry protocol. Drawing from existing pediatric ITP registries, this protocol encompasses the design of a minimum data set, patient exclusion criteria, information quality control, and follow-up procedures.

The pilot phase of this registry involved 60 pediatric ITP patients who were registered at Mofid Children’s Hospital, serving as the central registration center, along with our collaborative centers. While the literature reports a connection between ITP and certain infectious diseases, there is no link to coronavirus infection in this registry [22]. Furthermore, no correlation was found between the types of ITP and infection in this study. The findings also indicate that there was no significant association between sex, age, and the type of ITP or bleeding manifestations. A retrospective study on children with ITP, similar to our registry, found no statistically significant difference in gender between chronic and non-chronic ITP cases [23].

According to international guidelines, bone marrow biopsy is not recommended for confirming ITP, as it cannot provide definitive proof. However, in this registry, only 6.6% of patients underwent a bone marrow biopsy. The symptoms of ITP can vary widely, ranging from mild to severe, or even asymptomatic patients to severe hemorrhages from various areas of the body [24]. It should be noted that symptomatic bleeding is rare, except in severe cases of ITP. Common symptoms in children include gum bleeding, epistaxis, purpura, and easy bruising [25]. Petechiae, purpura, and ecchymosis were the most frequently reported bleeding manifestations in the NPITP registry. Lower platelet counts are associated with more severe conditions [26]. Newly diagnosed patients are typically treated with steroids, IVIG, and intravenous anti-D to improve their platelet counts. However, some patients do not respond to conventional first-line therapies. Based on the findings, most registered patients received altered doses of IVIG and corticosteroids. It is important to note that patients treated with corticosteroids often experience serious adverse events, including weight gain, diabetes, hypertension, emotional instability, sleep disorders, gastritis, thinning skin, and osteoporosis. In a study where patients with ITP were treated with corticosteroids, 98% experienced at least one side effect, and approximately 38% had to reduce or discontinue treatment due to adverse reactions [9]. Weight gain, emotional instability, and sleep disorders were the most commonly reported adverse events. Additionally, around 20% of ITP patients did not respond to treatment [27]. Several clinical trials have shown that a single administration of IVIG, up to 0.8 g/kg, is the most effective in increasing platelet counts compared to other IVIG doses [28].

Despite the valuable role of patient registries in enhancing the quality of care and clinical research, even a well-designed registry can have limitations and incomplete data collection. However, it is important to highlight the strengths of this registry. One significant strength of our study is that the results will contribute to the development of a national pediatric ITP guideline, aiming to reduce unnecessary diagnostic and therapeutic procedures and to be shared with other international registries.

## Conclusion

In conclusion, the pilot phase of the NPITP, serving as the first Iranian ITP registry, has successfully implemented a web-based software for data collection to enhance the quality of care, clinical research, and health services planning.

## Data Availability

The data that support the findings of this study are available at: https://www.researchgate.net/publication/373979571_The_First_National_Pediatric_Immune_Thrombocytopenic_Purpura_Registry_in_Iran_Research_Protocol_and_Preliminary_Study_Results.
